# Reply to: Erosive balanitis caused by *Staphylococcus haemolyticus* in a healthy, circumcised adult male

**DOI:** 10.1099/acmi.0.000765.v2

**Published:** 2024-02-14

**Authors:** Georgios Kravvas, Richard Watchorn, Christopher B. Bunker

**Affiliations:** ^1^​ Department of Dermatology, University College London Hospitals NHS Foundation Trust, London, UK

**Keywords:** lichen sclerosus, *Staphylococcus haemolyticus*, phimosis, balanitis, balanoposthitis

## Abstract

In this short letter of correspondence, we provide our specialist interpretation of what has been described in a previously published case report. We argue that this case describes a patient with chronic, undertreated male genital lichen sclerosus. If left unchecked, as in this case, lichen sclerosus can cause permanent architectural changes and damage to the affected tissues, and can thus predisposes to secondary infections, including bacterial, such as with *Staphylococcus haemolyticus*.

## Data Summary

This work has not generated any new data.

## Main text

Sir, our attention has recently been brought to this case report. Our interpretation of what has happened to this patient differs from that of the authors. The history and the signs suggest chronic, destructive, and undertreated lichen sclerosus (LSc), managed only partially by posthectomy. Unfortunately, no clinical photographs have been provided of the preoperative state of the penis, and no mention has been made of the presence or absence of preputial constriction or phimosis. We are told that he has suffered ‘several episodes of balanoposthitis in the 3 years’ preceding circumcision. Although balanoposthitis can occur for several reasons, the commonest being LSc, it is not in itself a diagnosis. A large body of evidence shows that LSc is due to chronic exposure of susceptible epithelium to urinary occlusion by the foreskin, or on some occasions by a ‘neo’ or ‘pseudo’ foreskin, as intimated here [1, 2]. The epithelial damage consequent upon chronic LSc will predispose to all manner of infections, including candida, herpes, staphylococci, and streptococci, as well as more exotic or rare microorganisms [3, 4]. This may well be the first time *Staphylococcus haemolyticus* has been isolated from a penis and that is of course worth describing and publishing, but we postulate is that this is not a primary infection, but rather infection complicating chronically damaged genital epithelium attributable to LSc. Yes, the circumcised male described here was healthy overall, but the genital epithelium was not.
